# Effective Classification and Gene Expression Profiling for the Facioscapulohumeral Muscular Dystrophy

**DOI:** 10.1371/journal.pone.0082071

**Published:** 2013-12-13

**Authors:** Félix F. González-Navarro, Lluís A. Belanche-Muñoz, Karen A. Silva-Colón

**Affiliations:** 1 Instituto de Ingeniería, Universidad Autónoma de Baja California, Mexicali, México; 2 Computer Science Faculty, Universitat Politècnica de Catalunya, Barcelona, Spain; University of Valencia, Spain

## Abstract

The Facioscapulohumeral Muscular Dystrophy (FSHD) is an autosomal dominant neuromuscular disorder whose incidence is estimated in about one in 400,000 to one in 20,000. No effective therapeutic strategies are known to halt progression or reverse muscle weakness and atrophy. It is known that the FSHD is caused by modifications located within a D4ZA repeat array in the chromosome 4q, while recent advances have linked these modifications to the *DUX4* gene. Unfortunately, the complete mechanisms responsible for the molecular pathogenesis and progressive muscle weakness still remain unknown. Although there are many studies addressing cancer databases from a machine learning perspective, there is no such precedent in the analysis of the FSHD. This study aims to fill this gap by analyzing two specific FSHD databases. A feature selection algorithm is used as the main engine to select genes promoting the highest possible classification capacity. The combination of feature selection and classification aims at obtaining simple models (in terms of very low numbers of genes) capable of good generalization, that may be associated with the disease. We show that the reported method is highly efficient in finding genes to discern between healthy cases (not affected by the FSHD) and FSHD cases, allowing the discovery of very parsimonious models that yield negligible repeated cross-validation error. These models in turn give rise to very simple decision procedures in the form of a decision tree. Current biological evidence regarding these genes shows that they are linked to skeletal muscle processes concerning specific human conditions.

## Introduction

The Facioscapulohumeral Muscular Dystrophy (FSHD) is an autosomal dominant neuromuscular disorder and the third most common inherited muscular dystrophy [1], [2]. Its incidence may vary in different places and probably in different racial groups, but recent estimates account for one in about 400,000 to one in 20,000 [3]. FSHD patients show progressive weakening and atrophy of the muscles in the face, slowly progressing to the shoulder, upper arm muscles and shoulder girdle, down to the stomach and lower limbs. Inability to flex the foot upward, foot weakness, and an onset of right/left asymmetry are also common symptoms [4], [5].

Although the FSHD is considered a relatively benign dystrophy, about 20% of the patients presenting this disorder are eventually restrained to a wheel chair. The age of onset is variable, being the second decade of life the most common stage where patients become symptomatic. In some cases, however, symptoms never develop even when the individual has the mutation associated with the FSHD.

No effective therapeutic strategies are known to either halt progression or reverse muscle weakness and atrophy in the FSHD [6]. However, there are a number of actions that can provide symptomatic and functional improvement in many patients. In particular, the use of assistive devices –such as braces, standing frames, or walkers– is of great help. Physical therapies like exercises in water, complemented by psychological support and speech therapy may also alleviate specially difficult life conditions.

It is known that the FSHD is caused by deletion of a subset of D4Z4 macrosatellite repeat units in the subtelomere of chromosome 4q [7]. D4Z4 modification needs to occur on a specific chromosomic background to cause the FSHD. More than 95% of patients with clinical FSHD have an associated D4Z4 deletion on the 4q35 chromosome. However, a small number of kindreds with clinically typical FSHD do not present this dynamic. A second FSHD locus has not yet been identified [8]. Recent advances involve the *DUX4* gene, a retrogene sequence within D4Z4 that encodes a double homeodomain protein whose exact function is not entirely known. Although the proper mechanisms responsible for the progressive muscle weakness still remain unknown, the study of this gene could offer a possible therapeutic way [7].

It is generally believed that the monitoring of expression levels for thousands of genes simultaneously may lead to a more complete understanding of the molecular variations among different cell conditions. In the literature on machine learning, contributions concerning the analysis of gene expression FSHD data are very scarce, probably because of unawareness towards the highly rare diseases. The situation is aggravated by the absence of scientific data outside purely medical domains, in order to attack the problem from a different point of view. In contrast, there is now a vast body of available datasets about microarray gene expression analysis when focused to *cancer* diseases. Specifically, microarray gene expression databases have been used to discriminate between tumours or tumour subtypes, and to study biological properties of tumours –see, e.g., [9].

Over the last decade, Machine Learning (ML) has made significant inroads in the fields of bioinformatics and biomedicine [10]. Specifically, cancer research has applied a variety of ML algorithms for tumor prediction by associating expression patterns with clinical outcomes for patients with tumors [11]. The majority of this research has focused on building accurate classification models from reduced sets of features. Some of these analyses also aim to gain understanding of the differences between normal and malignant cells and to identify genes that are differentially regulated during cancer development. The importance of the validity and reproducibility of statistical analysis and reporting cannot be stressed enough [12].

Typically, a gene expression data set may consist of dozens of samples but with thousands or even a few tens of thousands of genes (acting as *features*, using the ML terminology). Predictive model construction using this very high ratio between number of features and number of samples is a delicate undertaking, prone to obtain unreliable readings. As a result, dimensionality reduction and in particular *feature selection* techniques may be very useful, as a way to reduce the problem complexity and lighten medical expert diagnosis.

Of special importance in a practical medical setting is the *interpretability* of the obtained solutions, something that limits the applicability of methods such as PCA or ICA (whose solutions involve weighted combinations of genes, instead of individual genes). Moreover, in a medical context, data visualization in a *low-dimensional* representation space may become extremely important, as it would help doctors to gain insights into this complex and highly sensitive domain. The development of predictive models able to discern between healthy and FSHD samples with minimal error rate and amenable to direct interpretation is thus a clear research goal. When predictive models use very low numbers of relevant genes, these genes are likely to be associated with the disease, and can be used as a starting mechanism for further dedicated study from a biological point of view.

The present study addresses all these issues in two FSHD databases (named, just for reference in this paper, as FSHD-DB1 and FSHD-DB2) to discern between healthy and FSHD samples (clinical cases). We report experimental results supporting the practical advantage of combining robust feature selection and classification in the analyzed FSHD datasets. The described method is able to unveil two groups of genes that yield very low mean cross-validation error. These genes can be used to build very simple decision procedures in the form of a decision tree.

## Results and Discussion

### FSHD-DB1 Database

The feature selection process in **Algorithm 1** comes to a final solution in the form of a subset with only three genes and a 100% of mean 5×5 cv accuracy. This final subset is presented in Table 1 including its gene IDs and full names. It will be hereafter referred as the FSHD-DB1 model. In comparison, PAMR delivers a 96.8% of mean 5×5 cv accuracy with 2 genes (Table 2), and SVM-RFE delivers a comparable 99.4% mean 5×5 cv accuracy, using 5 genes (Table 3). As a further comparison, if we consider the two genes signaled as most relevant in the literature (*DUX4* [7] and *FRG1* [13]), the corresponding mean 5×5 cv accuracy of these two genes (taken together) is 84.65%.

**Table 1:** Best gene subset found using the proposed method and LDA as performance measure in FSHD-DB1 (the FSHD-DB1 model).

Probe set ID	Gene	Name
201088_*at*	KPNA2	karyopherin alpha 2 (RAG cohort 1, importin alpha 1)
219746_*at*	DPF3	D4, zinc and double PHD fingers, family 3
201552_*at*	LAMP1	lysosomal-associated membrane protein 1

**Table 2:** Best gene subset found using PAMR in FSHD-DB1.

Probe set ID	Gene	Name
218959_*at*	HOXC10	homeobox C10
215000_*s*_*at*	FEZ2	fasciculation and elongation proteinzeta 2 (zygin II)

**Table 3:** Best gene subset found using SVM-RFE in FSHD-DB1.

Probe set ID	Gene	Name
202594_*at*	LEPROTL1	leptin receptor overlapping transcript-like 1
208065_*at*	ST8SIA3	ST8 alpha-N-acetyl-neuraminide alpha-2,8-sialyltransferase 3
209797_*at*	CNPY2	canopy 2 homolog (zebrafish)
215000_*s*_*at*	FEZ2	fasciculation and elongation protein zeta 2 (zygin II)
218959_*at*	HOXC10	homeobox C10

#### Visualization

**Figure 1:**
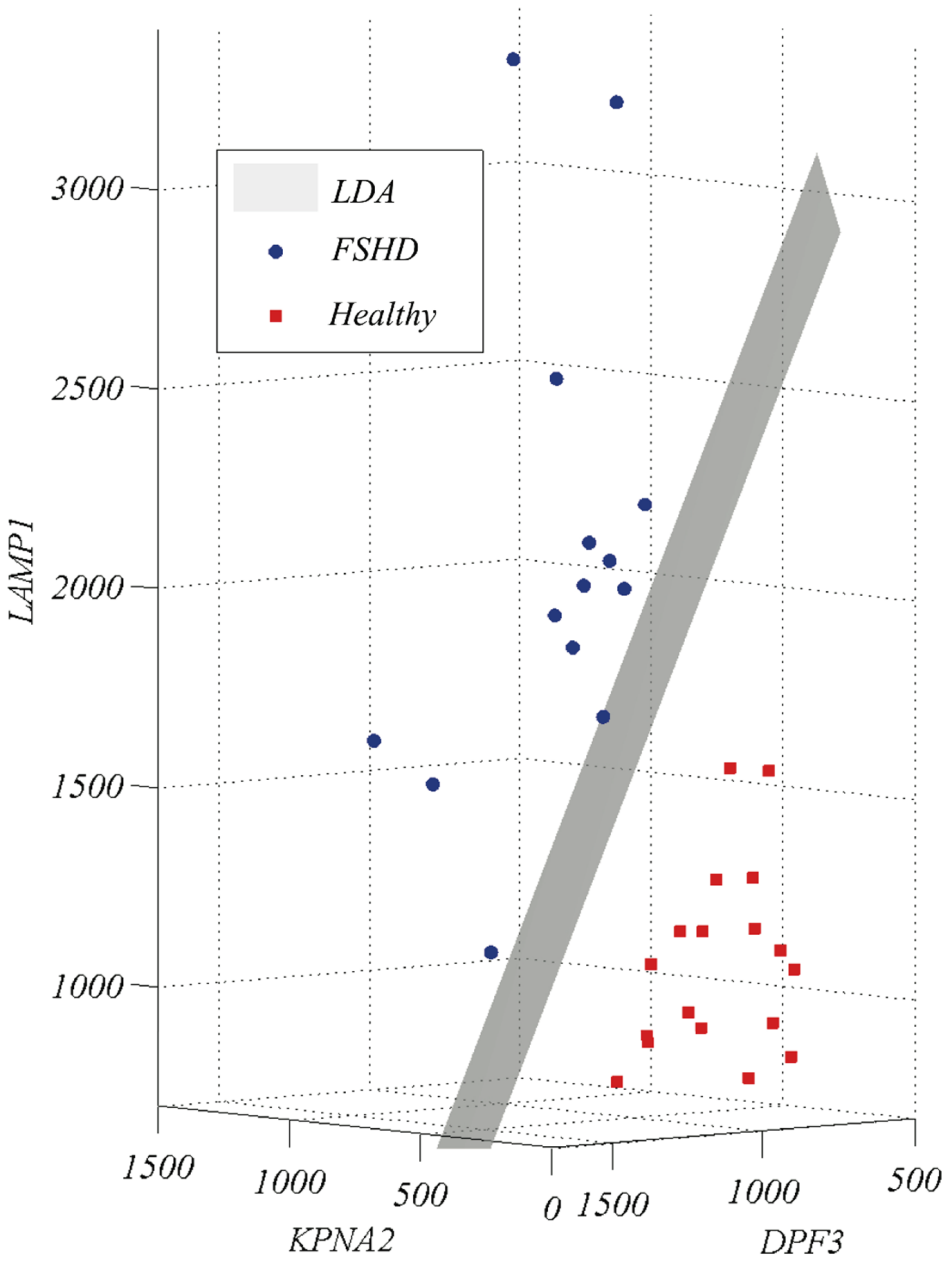
LDA decision surface for the FSHD-DB1 model.

Data visualization in a low-dimensional representation space is extremely important to gain a better understanding of the solution delivered by the process. To visualize the result, the data corresponding to the FSHD-DB1 model are plotted using the three selected genes as axes, without any pre-processing method or projection technique –Fig. 1. In addition, the LDA decision boundary fitted in the whole data set is shown. The FSHD group presents a less compact distribution, while the Healthy group is clustered around a specific region of the representation space given by the three genes found. It can be seen that the two conditions are neatly separated.

**Figure 2:**
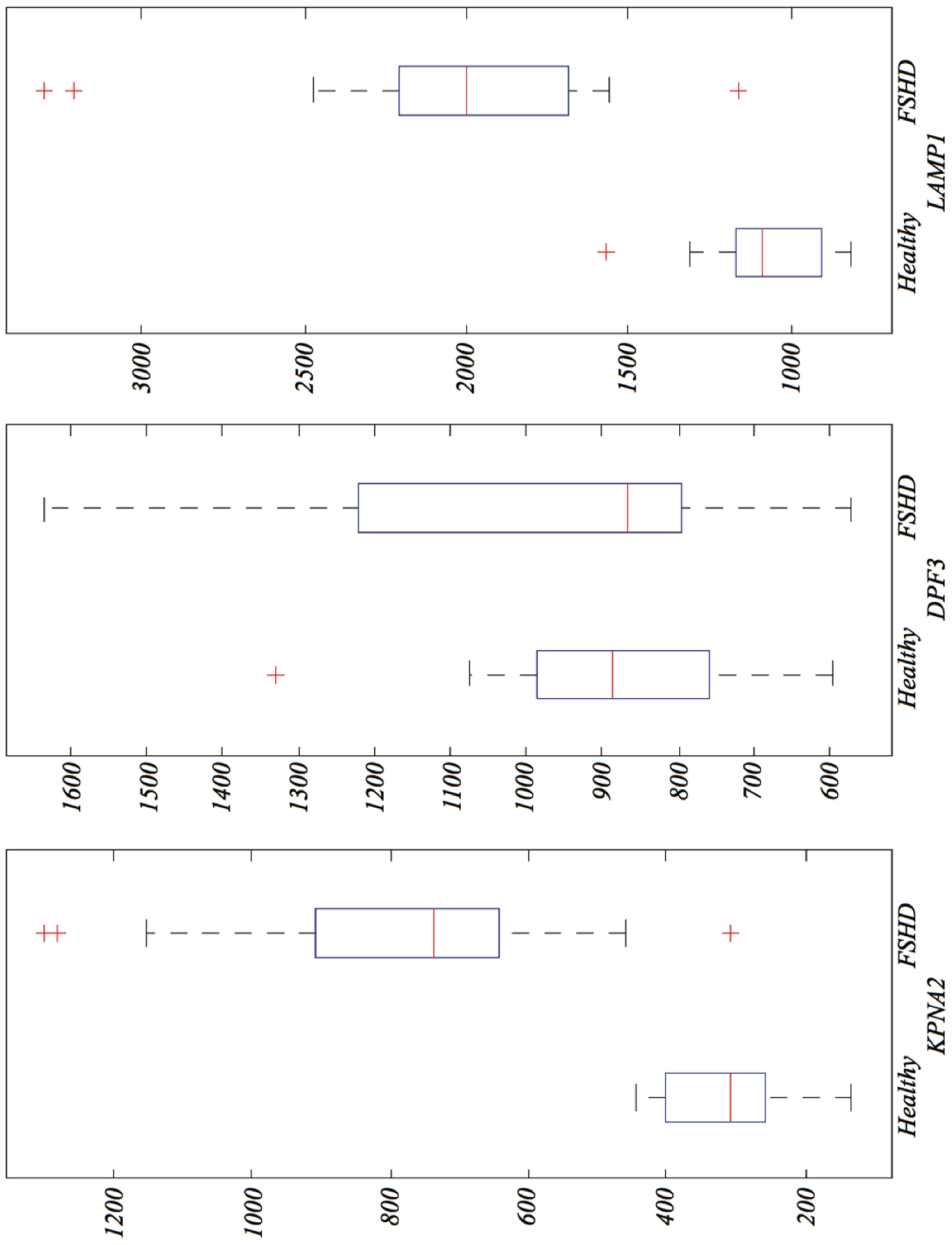
Box plots for the expression levels of the genes in the FSHD-DB1 model.

Figure 2 shows a box plot for each gene in the FSHD-DB1 model. *LAMP1* shows a mean value for FSHD samples of 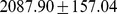, against Healthy with mean 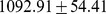; *DPF3* shows a more even expression level, FSHD with 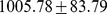 and Healthy with 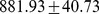; *KPNA2* tends to up-regulate heavily in FSHD (mean 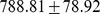, compared to Healthy with 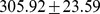).

**Figure 3:**
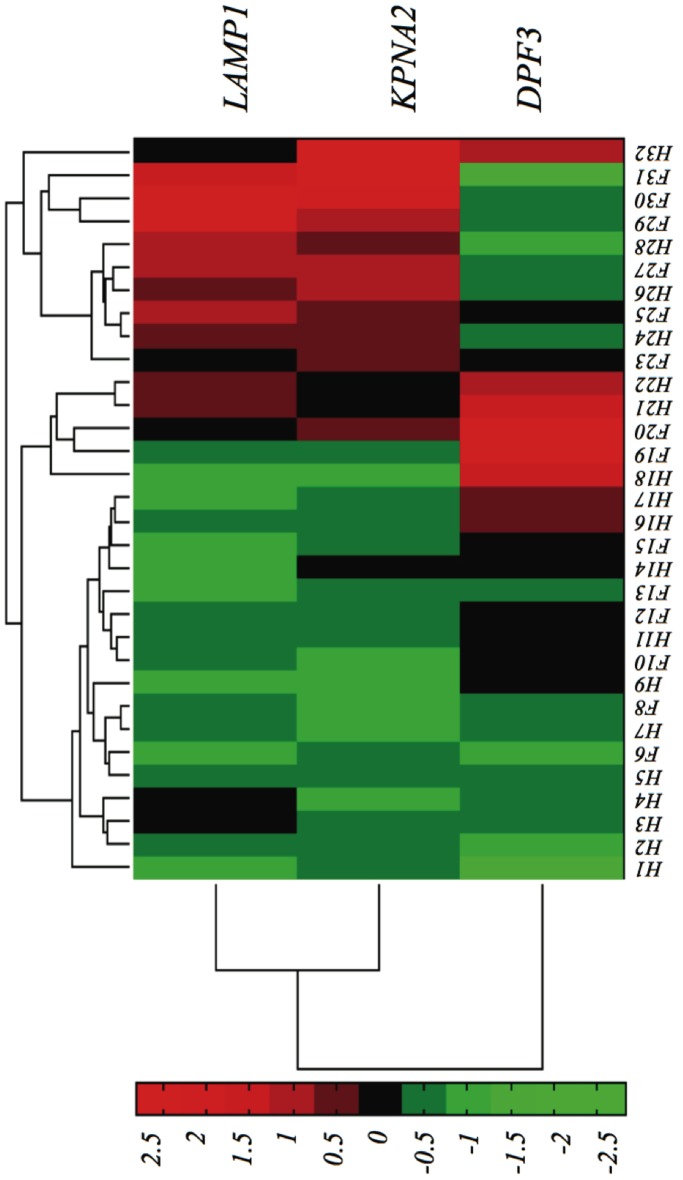
Clustering of the expression levels of the genes in the FSHD-DB1 model. Left: by genes; Top: by samples.

Figure 3 depicts a dendrogram of cases and standardized gene expression levels for the FSHD-DB1 model. Each case is identified with an ID number, prefixed by a letter indicating class membership, *H* for Healthy and *F* for FSHD. It is apparent that *LAMP1* shows an up-regulation in most of the FSHD cases, as well as *KPNA2*; *DPF3* shows a slightly diffuse expression level.

Nonetheless, it is noticed in Fig. 3 that the natural clusters do not necessarily correspond to labeled samples, and thus supervised information is needed to create accurate prediction models, even in this low-dimensional representation. Three clusters are discovered: a first one (H1 to H17), in which most (but certainly not all) of the samples belong to Healthy class; a second group (H18 to H22), containing three Healthy and two FSHD samples; finally, a third group (from F23 on) which is completely messed up. This result –although is certainly dependent on the limitations of clustering methods– alerts against using unsupervised feature extraction methods like PCA.

An interesting point to be emphasized in these graphic representations is that the FSHD-DB1 model clearly clusters the two conditions neatly –Fig. 1. We were therefore interested in ascertaining to what extent is this result stable and may thus constitute a good departing point for future studies. To this end, we performed two further investigations:

1. The first action was to change the resampling method to 10 times 10-fold cross validation (10×10 cv). This form of resampling entails a much higher computational cost; however, it has been suggested as adequate for small sample situations [14].
2. The second action was to analyze the statistical differences between FSHD vs. Healthy samples in the expression levels for the genes in the model. In addition, we explored the possibility that a single gene is able to (almost) perfectly separate the two classes by mere chance.

#### Statistical analysis

We were interested in exploring the effect of changing the resampling method, keeping the *same* classifier (LDA in this case), in order to exclude this source of variation from the analysis. Remarkably, using 10×10 cv instead of 5×5 cv in **Algorithm 1**, it was found that the final result fully coincided with the FSHD-DB1 model.

In order to assess statistical significance of expression levels, the Mann-Whitney U-test (MWU) was used in the comparison between FSHD vs. Healthy samples in the model. This is a non-parametric hypothesis test for assessing whether one of the two conditions (FSHD in this case) tends to have larger values than the other.

For *KPNA2*, medians for the two groups Healthy and FSHD were 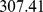 and 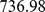; the distributions in the two groups differed significantly (Mann-Whitney W = 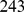, 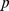-value 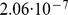).

For *DPF3*, medians for the two groups Healthy and FSHD were 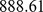 and 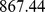; the distributions in the two groups did *not* differed significantly (Mann-Whitney W = 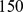, 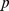-value 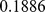).

For *LAMP1*, medians for the two groups Healthy and FSHD were 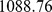 and 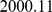; the distributions in the two groups differed significantly (Mann-Whitney W = 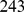, 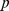-value 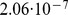).

Therefore both *KPNA2* and *LAMP1* genes present high differences in the two conditions. Although these two genes are not equal, they present notable similarities. Spearman’s rank correlation coefficient is equal to 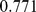. This fact will be used to simplify the FSHD-DB1 model.

One may still wonder about the probability of finding such a single gene like *KPNA2*–that separates the two conditions with one exception– by mere chance (Fig. 2). If a gene bears no relation with the disease, we could expect an arbitrary pattern for the distribution of the two conditions (healthy vs. FSHD cases) across the expressed values of the gene. The probability that *one or more* genes in 22,283 separates the two conditions (14 FSHD; 18 healthy) with only one exception is found to be around 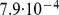.

#### A final interpretable model

Even though the LDA decision boundary in Fig. 1 depicts a clean separation between the two patient conditions, its application as a decision tool may not be straightforward. In this sense, decision trees are one of the preferred tools by experts in decision making processes. Moreover, the final selection of a gene subset may still provide few clues about the structure of the two conditions with respect to their expression levels. Some accuracy may be sacrificed for increased interpretability of the model.

**Figure 4:**
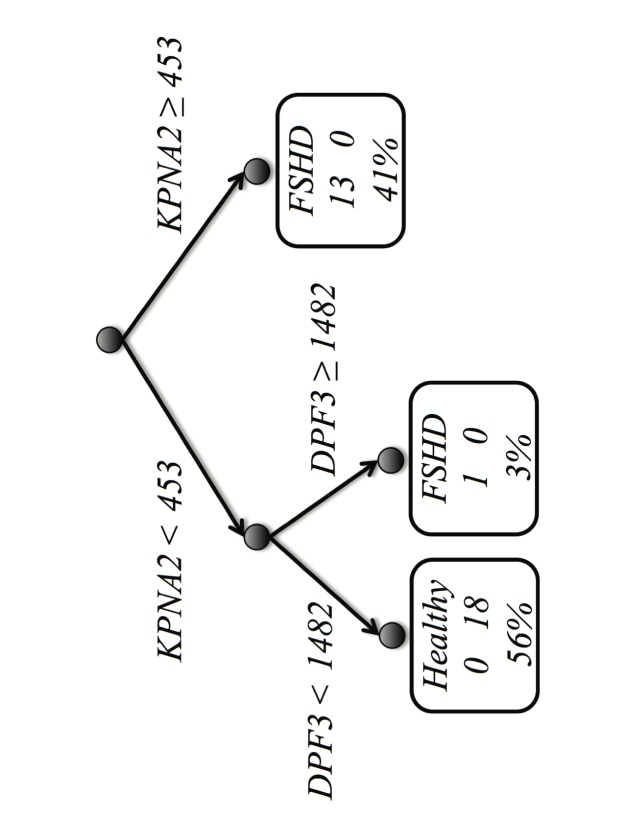
Classification tree for the simplified model in the FSHD-DB1 database. The boxes are leaves indicating the prediction, the numbers of cases for each condition, and the overall percentage of covered cases.

Figure 4 shows a CART decision tree [15] built with the FSHD-DB1 model. The main question is on the expression level of gene *KPNA2*: the right branch corresponds to 13 (all but one) of the FSHD patients; the left branch corresponds to all of the 18 healthy ones plus the remaining FSHD patient. Moreover, one may wonder if there is a second gene, expressed such that it separates this specific patient from the 18 healthy ones, and indeed there is one: precisely *DPF3*. Whether this last patient is an outlier in a medical sense we cannot know, but it deserves further clinical investigation. Therefore, despite *LAMP1* shows a markedly differential expression, it may be excluded from the decision flow.

#### Biological evidence

In this section, we compile scientific knowledge about the two genes in the final subset, including their known primary functions in cellular process.

#### KPNA2

*KPNA2* is Karyopherin alpha 2 (RAG cohort 1, importin alpha 1). It is known that muscle functions are dependent on spatial and temporal control of gene expressions in *myofibers*. These are multinucleated cells that contain hundreds of nuclei spread across the length of the cell in a common cytoplasm. Their very important role is to control the transcriptional activity of several nuclei in a common cytoplasm [16].

Analyzing the role of karyopherin alpha (KPNA) and paralogs-specific roles of *KPNA1* and *KPNA2* during myogenesis, it has been found that these two genes do regulate myoblast proliferation. Particularly, *KPNA2* regulates myotube size and myocyte migration [17]. Therefore, both may be involved in the nuclear transport of proteins [18], which has a key role in controlling gene expression in skeletal muscles.

#### DPF3

*DPF3* is D4, zinc and double PHD fingers, family 3. This gene belongs to the neuron-specific chromatin remodeling complex (nBAF complex), acting as a tissue-specific anchor between histone acetylations and methylations and chromatin remodeling [18], [19]. Experiments in human cardiac samples and mouse embryonic and adult hearts showed that it plays a role in heart and skeletal muscle development [20]. It also presents an up-regulated expression in patients with *Tetralogy of Fallot*, a congenital heart defect, partially characterized by muscular hypertrophy.

### FSHD-DB2 Database

The feature selection process in **Algorithm 1** comes to a final solution with six genes and 99.6% of mean 5×5 cv accuracy. This final subset is presented in Table 4 including its gene IDs and full names (of which two of them are yet unknown). It will be hereafter referred as the FSHD-DB2 model. In comparison, PAMR delivers a 70.4% of mean 5×5 cv accuracy with 3 genes (Table 5), and SVM-RFE delivers 85.2% mean 5×5 cv accuracy, using 5 genes (Table 6, of which three of them are unknown). This database contains *DUX4* entries, corresponding to 4 isoforms. If we consider the most informative model, including the 4 sequences of *DUX4* and *FRG1* together, the corresponding 5×5 cv accuracy is found to be a disappointing 39.60%.

**Table 4:** Best gene subset found using the proposed method and LDA as performance measure in FSHD-DB2 (the FSHD-DB2 model).

Probe set ID	Gene	Name
7905039	Unknown	
8111670	GDNF	glial cell derived neurotrophic factor
7899075	EXTL1	exostoses (multiple)-like 1
7947152	RPL36AP40	ribosomal protein L36a pseudogene 40
7942073	IGHMBP2	immunoglobulin mu binding protein 2
8147750	Unknown	

**Table 5:** Best gene subset found using PAMR in FSHD-DB2.

Probe set ID	Gene	Name
8111892	OXCT1	3-oxoacid CoA transferase 1
8062461	LBP	lipopolysaccharide binding protein
8111670	GDNF	glial cell derived neurotrophic factor

**Table 6:** Best gene subset found using SVM-RFE in FSHD-DB2.

Probe set ID	Gene	Name
7893282	Unknown	
8129666	SLC2A12	solute carrier family 2 (facilitated glucose transporter), member 12
7926818	Unknown	
8094938	NIPAL1	NIPA-like domain containing 1
7938667	Unknown	glial cell derived neurotrophic factor

#### Visualization

**Figure 5:**
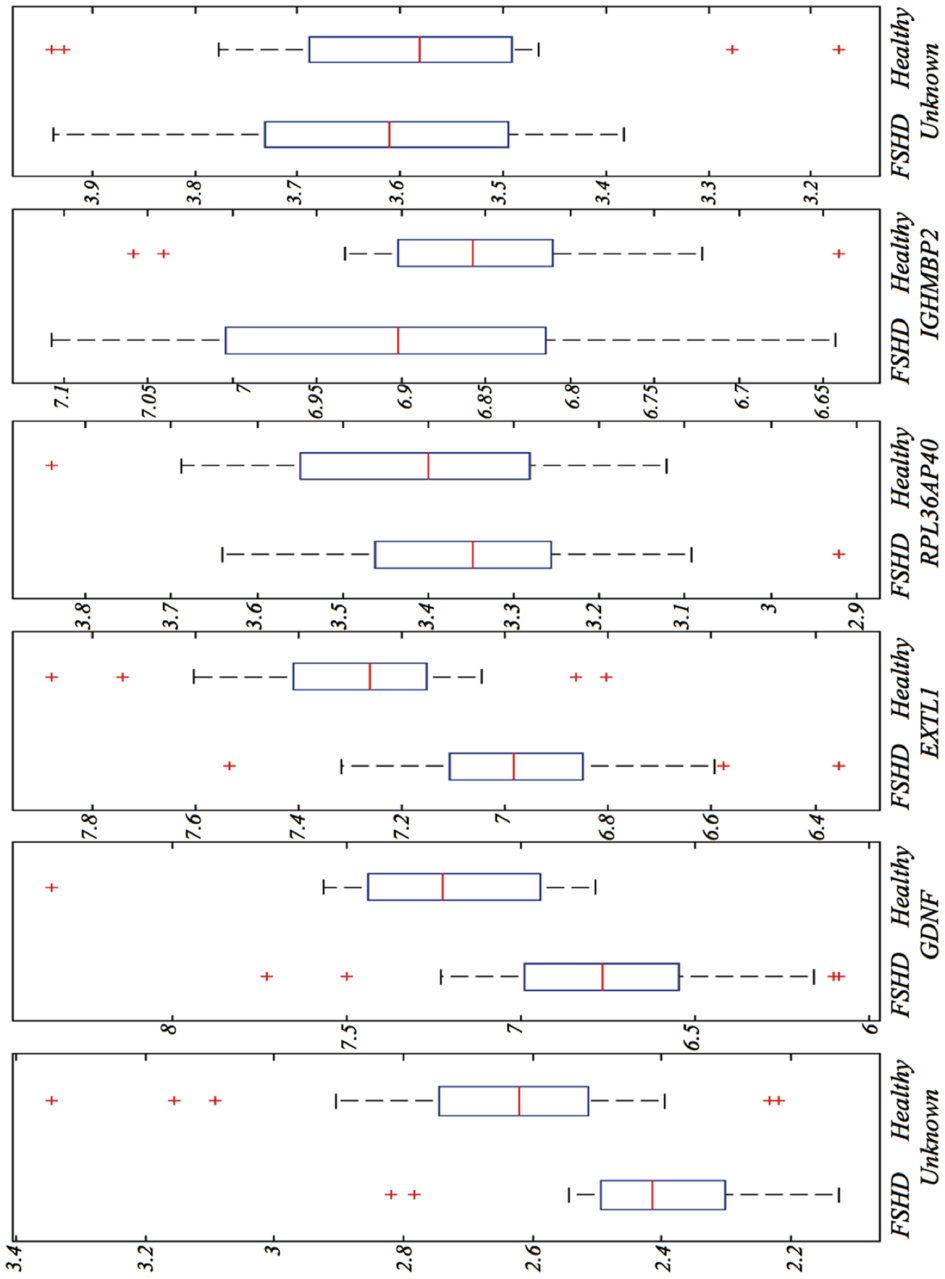
Box plots for the expression levels of the genes in the FSHD-DB2 model.

**Figure 6:**
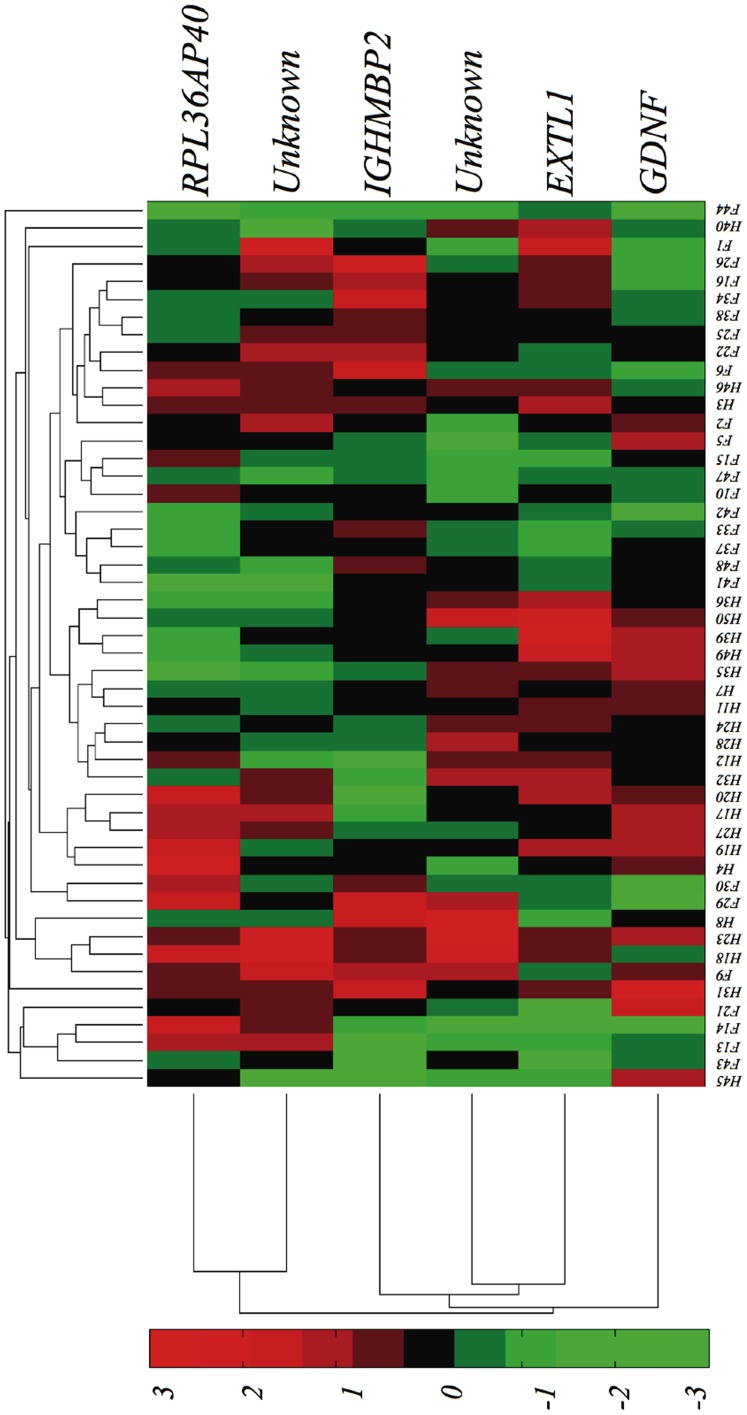
Clustering of the expression levels of the genes in the FSHD-DB2 model. Left: by genes; Top: by samples.

Figure 5 shows a box plot for each gene in the FSHD-DB2 model. The first three genes in the model (*Unknown-7905039*, *GDNF* and *EXTL1*) tend to up-regulate heavily, this time in Healthy samples. The other three seem to contain complementary information in the variance rather than in the central tendency. Figure 6 depicts a dendrogram of cases and standardized gene expression levels for the FSHD-DB2 model. Each case is identified with an ID number, prefixed by a letter indicating class membership, *H* for Healthy and *F* for FSHD. It is apparent that the natural clusters are less homogeneous than those obtained for the FSHD-DB1 database. Nonetheless, the group of central clusters (formed only by Healthy cases, H4 to H36) is clearly identified by *GDNF* and *EXTL1*, both genes showing a definite up-regulation in all cases.

#### Statistical analysis

Again, statistical significance of individual expression levels in the FSHD-DB2 model is assessed with a Mann-Whitney U-test (MWU) in the comparison between FSHD vs. Healthy samples.

For *Unknown-7905039*, medians for the two groups (FSHD and Healthy) were 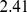 and 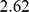, resp.; the distributions in the two groups differed significantly (MannWhitney W = 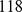, 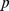-value 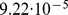).

For *GDNF*, medians for the two groups (FSHD and Healthy) were 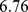 and 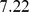, resp.; the distributions in the two groups differed significantly (MannWhitney W = 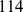, 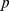-value 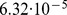).

For *EXTL1*, medians for the two groups (FSHD and Healthy) were 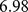 and 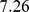, resp.; the distributions in the two groups differed significantly (MannWhitney W = 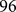, 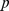-value 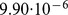).

For the other three genes, the medians for the two groups are very close and the test is non-significant at the 95% level. This seems to confirm the previous interpretation of a first subgroup of three genes (*Unknown-7905039*, *GDNF* and *EXTL1*) that contain highly discriminant information in their means (or medians) and a second subgroup of another three genes (*RPL36AP40*, *IGHMBP2* and *Unknown-8147750*) that complement the first group. Interestingly, this split fully coincides with the order in which the genes were discovered by the feature selection process in **Algorithm 1**. The second-ranked gene, *GDNF*, is also chosen by the PAMR method (Table 5).

In contrast to the previous database, the genes in the FSHD-DB2 model seem quite different and, this time, no single gene can separate the two conditions neatly; rather, they collaborate to reach a very high classification accuracy. Indeeed, the absolute value of Spearman’s rank correlation coefficient is lower than 0.5 in all cases, and specially low in the first subgroup of relevant genes.

#### A final interpretable model

**Figure 7:**
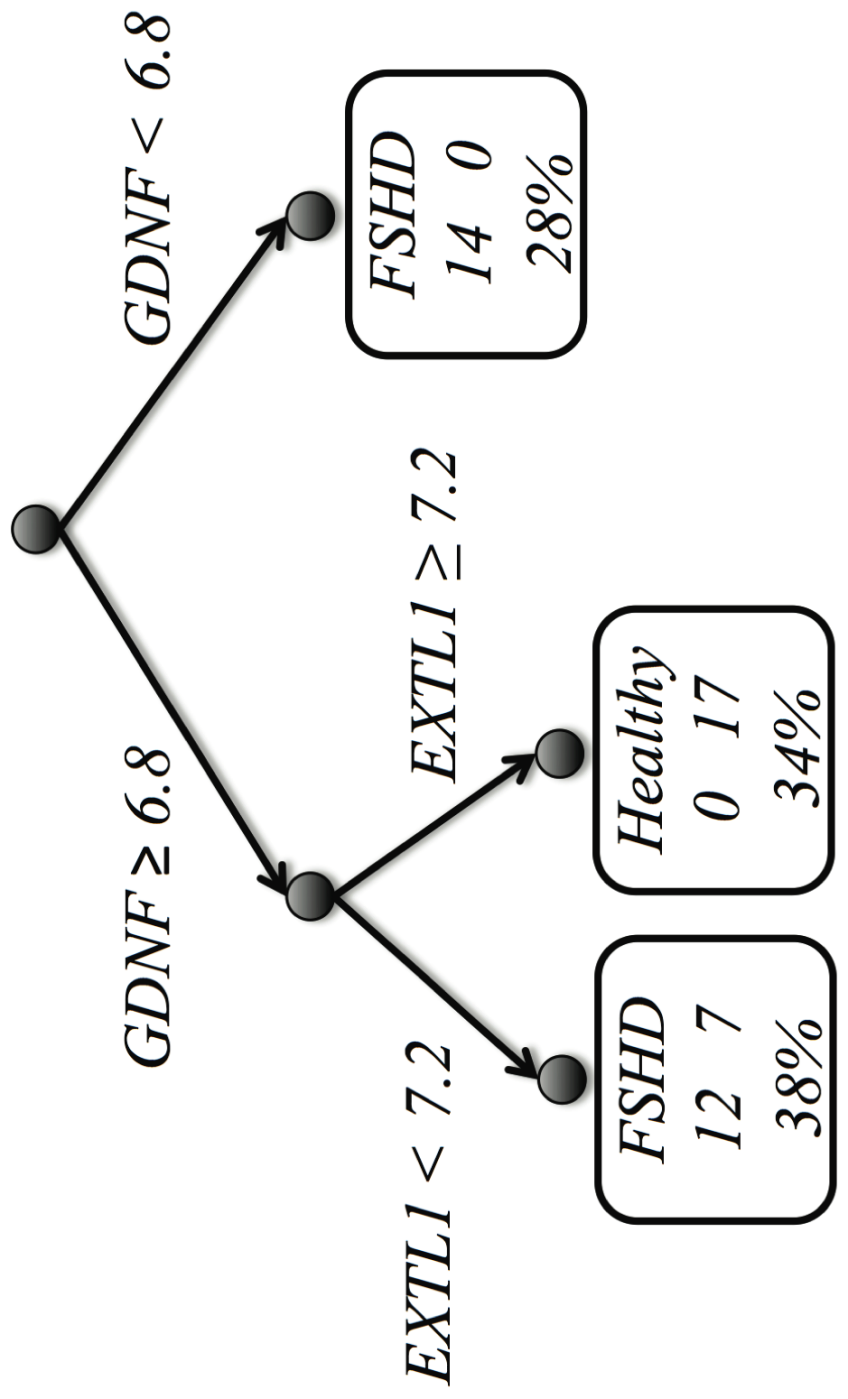
Classification tree for the simplified model in the FSHD-DB2 database. The boxes are leaves indicating the prediction, the numbers of cases for each condition, and the overall percentage of covered cases.

As for the previous database, accuracy may be sacrificed for increased interpretability of the model. Figure 7 shows a CART decision tree built with the FSHD-DB2 model. The interpretation of the tree is as follows: patients showing a value of *GDNF* lower than 6.8 are all classified (correctly) as having the FSHD condition, and this group constitutes 28% of the total; patients showing a value of *GDNF* greater than 6.8 and a value of *EXTL1* greater than 7.2 are all classified (correctly) as *not* having the FSHD condition, and this group constitutes 34% of the total; for the final group (38% of the total), 12 patients are correctly identified as having the FSHD condition, and the remaining 7 are incorrectly identified as having the FSHD condition; thus the tree makes 7 false positives and no false negatives.

#### Biological evidence

In this section, we compile scientific knowledge about the two genes in the final subset, including their known primary functions in cellular process.

#### GDNF

*GDNF* is *glial cell derived neurotrophic factor*: a gene encoding a highly conserved neurotrophic factor. The recombinant form of the protein has been shown to promote the survival and differentiation of dopaminergic neurons in culture, and is able to prevent apoptosis of motor neurons induced by axotomy [18]. GDNF is also associated to the Hirschsprung disease (HSCR), a congenital disorder typically characterised by a part or all of the large intestine having no nerves and intestinal obstruction, due to an absence of intramural ganglia along the intestine [21].

#### EXTL1

*EXTL1* is *exostoses (multiple)-like 1*. This gene is a member of the multiple exostoses (EXT) family of glycosyltransferases. The encoded protein is involved in chain elongation of some acidic complex polysaccharides found on the cell surface and in the extracellular matrix [18]. Mutation in EXT1 is associated with hereditary multiple exostoses, a human disorder characterized by the formation of cartilage-capped bony outgrowths at the epiphyseal growth plates [22].

## Concluding Remarks

The Facioscapulohumeral Muscular Dystrophy, or FSHD, is a highly rare muscle disease for which there is no known cure nowadays. Two databases presenting samples of both healthy and FSHD patients have been analyzed with machine learning (ML) methods. There is hardly any precedent in the literature addressing this disease with these techniques.

The fact that the FSHD data analyzed in this study are scarce and of high dimensionality makes their computer-based automated classification a difficult undertaking. Most importantly, this high dimensionality precludes a straightforward interpretation of the obtained results, limiting their usability in a practical medical setting. In this vein, computational solutions like the one reported here should reckon the need of reporting not only highly accurate models: they should also represent low complexity and interpretable solutions amenable to further analysis by experts.

We have devised an approach to prediction of the FSHD condition from gene expression profiling, comprising an effective algorithm for gene selection enhanced with a mechanism for tie-breaking and based on a fairly standard classifier. To demonstrate its effectiveness, we show that the method was highly efficient in identifying two subsets of genes that best characterize each class. In both cases, the discrimination process is shown very conveniently as a two-question decision tree. We have also provided evidence for the statistical significance and stability of the result. Our method delivers highly interpretable solutions that are more accurate than competing methods. The technique is general and could be used in other similar scenarios.

However, in small sample scenarios, there is a high risk of overfitting the data: small samples will appropriately support only simple models with few parameters (acting as the coefficients of the features). Moreover, the use of a classifier having one or more hyper-parameters (these are parameters that the classifier cannot determine in its training process, and must be determined externally) is unaffordable, since this would require an additional resampling loop, for which there would almost be no data left. As a consequence, the determination of these parameters would be subject to a very high degree of uncertainty. We have selected Linear Discriminant Analysis (LDA) as the target classifier, using equal-covariance Gaussians to approximate class conditional probability densities. This choice corresponds to a linear, stable and parameter-free classifier. The LDA recognition rate was resampled using 5 times 5-fold cross validation.

One should bear in mind that the excellent reported results do not –by themselves– entail a medical solution to the disease, a situation that is faced by all statistical and ML solutions. On the contrary, a main goal of exploratory studies of this kind should be aimed towards understanding how the variables selected by the model fit in relation to prior knowledge from the medical domain.

## Materials and Methods

### The FSHD Databases

The first database used in this contribution was obtained from the EMBL-EBI repository of the European Bioinformatics Institute [23]. Specifically, the *Experiment E-GEOD-3307* uses the Affymetrix GeneChip Human Genome HG-133A and HG-U133B designs to analyse a range of muscle diseases for gene expression comparative profiling purposes. A total of 121 muscle samples of 11 muscle pathologies (plus several healthy samples) integrate the data: acute quadriplegic myopathy, juvenile dermatomyositis, amyotophic lateral sclerosis, spastic paraplegia, fascioscapulohumeral muscular dystrophy, Emery Dreifuss muscular dystrophy, Becker muscular dystrophy, Duchenne muscular dystrophy, calpain 3, dysferlin, and the FKRP using U133A and U133B array design. These are diseases with a extremely low incidence rate in the general population. The Facioscapulohumeral Muscular Dystrophy (FSHD), the targeted group in this work, consists of 14 FSHD samples and 18 healthy samples described by 22,283 genes or features (HG-133A version).

The second database was obtained from the GEO (Gene Expression Omnibus) repository, a publicly available site in the National Center for Biotechnology Information (NCBI). The *Experiment GSE36398*, “Transcriptional profiling in facioscapulohumeral muscular dystrophy to identify candidate biomarkers” is a very recent database containing FSHD information only. Using the Affymetrix Human Gene 1.0 ST Array, the experiment analyses RNA extracted from both biceps and deltoids of FSHD subjects (26 samples) and unaffected first-degree relatives (24 samples), rendering a dataset that consists of 50 samples, described by 33,297 genes or features [24].

There are no missing data in any of the two datasets; and both contain a mixture of positive and negative examples, necessary for learning. Moreover, in both cases the whole datasets were used.

### Linear Discriminant Analysis

Linear and quadratic discriminant analyses or LDA/QDA (Duda et al. 2001) are widely used parametric methods which assume that the class distributions are multivariate Gaussians. With LDA, all classes are assumed to have the same covariance matrix. QDA does not need such an assumption; however, the number of parameters to be estimated from the data available for each class is much higher, entailing lower statistical significance.

In both methods, classification is achieved by assigning an example to the class for which the posterior probability 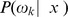 is greater, or equivalently for which 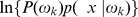 is greater.

These methods are attractive because they need no parameter tuning, and their limited complexity (quadratic at most) may be a solid guard against overfitting the data. Moreover, for LDA fast updating procedures exist for the computation of certain forms of the cross-validation error [25]. The *discriminant function* for class 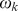 is expressed as:
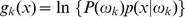


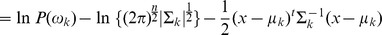
which simplifies to:
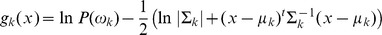



If we assume that all class-conditional distributions 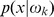 have the *same* covariance matrix 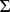, we get:
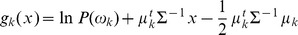



These are **linear discriminant functions** (linear in 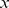) and the **decision boundaries** 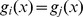 are hyperplanes in 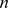-dimensional space.

In practical situations, only an i.i.d data sample 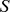 is available. When means, covariances and priors for every class are not available, maximum-likelihood estimates on 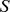 can be used, although in this case the Bayesian optimality properties are no longer valid. Let 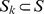 be the subset of samples known to belong to class 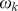. Then 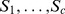 is a partition of 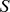. Unbiased estimates for the vector means and for the class priors can be obtained as:
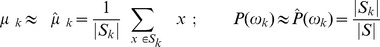



The following *pooled* covariance matrix is then used:
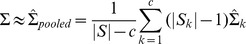
where
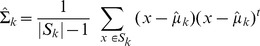



### Linear Support Vector Machines

The support vector machine (SVM) is a machine learning method solidly based on statistical learning theory [26]. Intuitively, given a set of examples labeled into one of two classes, the linear SVM finds their optimal linear separation: this is the hyperplane that maximizes the minimum orthogonal distance to a point of either class (this distance is called *margin* of the separation).

Consider again an i.i.d data sample 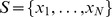 of training patterns (in 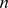 dimensions), labelled into two classes 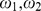 by 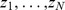, with 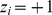 if 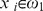 and 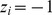 if 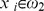. If we set up an affine function 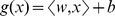, then we have a linear discriminant as 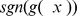, for which we would like:
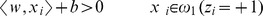


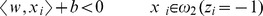



In short, 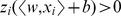, or 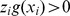, for all 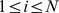. Given the hyperplane 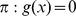, the perpendicular distance from 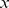 to 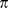 is 
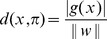
. The *support vectors* are those 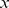 closest to the hyperplane. Rescaling 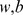 such that 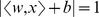 for these closest points, one obtains 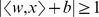. The *support vectors* are now those 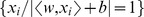.

The *margin* 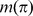 of a plane 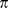 can now be written as twice its distance of any support vector: 
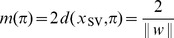
, where 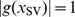. To maximize the margin, we should minimize 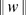 subject to 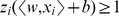, for all 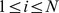.

In the case where an hyperplane does not exist that can separate correctly the points in the data sample, a set of non-negative *slack* variables are introduced to allow for small *margin violations*, leading to a *soft margin*:
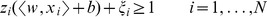
(1)where 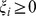. For an error to occur, the corresponding 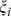 must exceed unity, and so 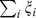 is an upper bound on the number of *training* errors. The optimal separating hyperplane can be found as the solution of the 1-norm Quadratic Programming problem:
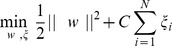


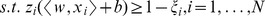



The solution to this optimization problem corresponds to the saddle point of its associated Lagrangian:
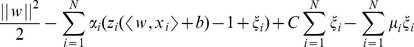
where 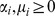 for 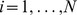.

Once this QP problem is solved, the solution vector 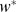 can be expressed as a linear expansion over the support vectors:
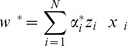
(2)


The support vectors are precisely those 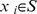 for which 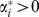.

### Resampling Methods

Model selection is concerned with the process of finding the optimal model for a set of samples among a set of candidate models. Resampling methods aim at making a better use of the available data. These methods are very useful for assessing how a predictive model that can be the result of a complex modeling process will perform in practice.

The generic goal of cross-validation (CV) is to estimate the expected error of a model in a data set that is independent of the data that were used to train the model. One round of 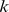-fold CV (or 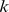-CV) involves partitioning the sample into 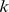 complementary subsets, systematically performing the modeling on the union of 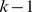 such subsets and checking the obtained model on the remaining subset (acting as a validation set). The result of 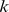-CV is an estimation of the error if only a fraction 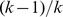 of the available data is used. This error is expected to be conservative (larger than the error obtained if the entire sample was used). To reduce variability, multiple rounds can be performed using different partitions, and the results averaged over the rounds.

### The Feature Selection Algorithm

Feature selection can be seen as a search problem, where each state in the search space corresponds to a subset of the features. In the ML literature, a wide family of suboptimal algorithms depart from an initial solution and iteratively add or delete features by locally optimizing the error function. In *forward selection*, features are progressively incorporated into larger subsets; in *backward selection* (or elimination) one starts with the full set of features and progressively eliminates elements from it.

Wrappers are often criticized because they are computationally very expensive. Moreover, feature selection is badly affected by small sample sizes, producing overly optimistic results and introducing an excess of variance in the readings. This is aggravated in the presence of very sophisticated search algorithms [27]. On the other hand, greedy search strategies seem to be particularly computationally advantageous and may alleviate the problem of overfitting [28]. Nevertheless, traditional pure forwardd selection and backward elimination search algorithms are ill-advised in that they cannot rectify their decisions and may end up delivering poor solutions both in terms of quality and size.

To reduce the number of genes and obtain small subsets of highly relevant genes, we use a simple but effective forward-backward feature selection algorithm. This algorithm follows the wrapper idea, *i.e.*, the feature selection algorithm uses a learner as a subroutine in the search for good subsets [29]. In this general setting, when features are added or removed from the current subset the algorithm resorts to some performance measure –commonly the resampled rate of recognition.

An interleaved forward-backward search is developed looking for the improvement in performance of the chosen performance measure. The algorithm is described as the listing **Algorithm 1**. Given a performance measure 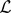 to be maximized (in this case, the resampled evaluation of a classifier in a data sample), the algorithm searches the space of subsets by adding/removing features in a hill-climbing fashion.

Specifically, in every iteration of the outer loop, one feature is added to the current best solution 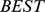, as long as this step improves on current performance 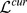. Then a variable number of feature removal steps is carried out, inasmuch the same condition of improved performance is met. This scheme is oriented to favour solutions with low numbers of features. The outer iteration also ends when no further improvement is observed. This strategy bears some resemblances with a floating search algorithm in its forward version [30]. However, it has a far lower computational cost given that discarded features are not considered again for another inclusion round. Note also that current subset performance is not compared specifically against the best performance achieved for the *same* size of the current subset (as floating methods do). It should be mentioned that the algorithm itself needs no parameter specification, although the chosen performance measure could have.

**Algorithm 1** Forward-Backward gene feature selection.

1: Input: 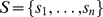: Full feature set;


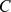
: Class feature (Healthy, FSHD)


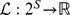
: performance measure, to be maximized

2: 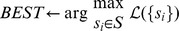


3: 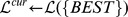


4: 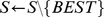


5: repeat

6: ***Forward Stage***

7: 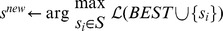


8: 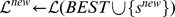


9: **if** 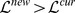 **then.**

10: 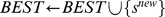


11: 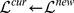


12: 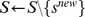


13: **end if**

14: ***Backward Stage***

15: repeat

16: 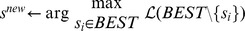


17: 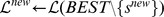


18: **if** 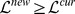 **then**

19: 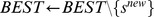


20: 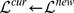


21: **end if**

22: **until** *BEST* **does not change**

23: **until** *BEST* **does not change**

24: **Output**: *BEST*: **Optimized feature subset**

As explained in the introduction, we are interested in a solution that combines high predictive performance, very small size (*i.e.*, a very low number of useful genes), admits visualization and interpretation, and hopefully may bear biological relevance.

To this end, we explicit now how the methods previously described glue together. The performance measure 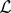 to be maximized in **Algorithm 1** is the accuracy rate of LDA. This recognition rate is resampled using 5 times 5-fold cross validation (5×5 cv for short), following common practices in the literature [31].

Due to the low number of samples, ties among the performance measure can happen easily. As a consequence, the gene subset selection process will end up in different final solutions, something that is not desirable in general [32]. How these ties are broken is non-trivial and should be addressed specifically and explicitly. However, the literature does not seem to offer any formal solution or procedure. Univariate methods as entropy-based measures [33], [34], the Fisher Score [35], or some other statistical measures could be those preferred for their simplicity –see *e.g.* [36], [37]. Instead, a *multivariate* feature ranking method seems much more adequate to measure the relevance of a group of tied features.

As explained above, linear support vector machines (SVMs) can be seen as linear discriminant classifiers. Indeed, the numbers 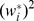 in eq. (2) have been used as a surrogate for the relevance of the 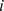-th gene since the pioneering work of [38]. Notice that our approach is different in that predictive performance is the main criterion for optimization. Only in case of ties is the magnitude of the SVM weight vector being used. This is because the relation between this magnitude and final performance is rather indirect. This margin-based *tie-breaking* procedure has been incorporated into the feature selection algorithm. It is used every time an evaluation of the performance measure may incur on one or more ties –lines 2,7 and 16 in **Algorithm 1**.

### Other Methods

#### Prediction analysis for microarrays

PAMR (Prediction Analysis for Microarrays) performs sample classification from gene expression data, via the nearest shrunken centroid method [39]. Similarly to the proposed method, PAMR estimates prediction error via cross-validation and provides a list of significant genes whose expression characterizes each diagnostic class.

#### Support vector machine for recursive feature elimination

SVM-RFE (Support Vector Machine - Recursive Feature Elimination) [38] has been used widely with great success in microarray data analysis, particularly for disease gene finding. It largely eliminates redundant genes and usually yields very compact gene subsets. The genes are eliminated according to a ranking related to weight magnitude in the SVM solution. This is the same criterion for tie-breaking described in the previous section.

### Software Implementation

**Algorithm 1** was implemented entirely in MATLAB language, version 2012a. The computer codes were run on an Ubuntu Linux server version 11.10 with an Intel(R) Xeon(R) CPU E5620 @ 2.40 GHz and 8 cores. The deployed solution to **Algorithm 1** takes advantage of the possibility to parallelize parts of the code, particularly lines 2, 7 and 16. In an 8-core scenario, eight genes or features can be evaluated at the same time. The complete software and instructions to reproduce the experiments described in this paper (or to conduct new ones) is available at http://nova.mxl.uabc.mx/fernando/PO/for the interested reader.

The LDA classification algorithm and the resampling methods implied in **Algorithm 1** are developed using already existing MATLAB functions. The only part that uses an external toolbox is in the tie-breaking procedure –eq. (2). The well-known Steve Gunn’s MATLAB Support Vector Machine Toolbox [40] was used for this purpose. Full specification of parameters is described in the url link given above.

It is important to clarify that the data sets were used without any pre-processing step. The learning algorithms and the complete experimental setting were fed with the original downloaded E-GEOD-3307 and GSE36398 data. Complete details about the E-GEOD-3307 data set can be found at http://www.ebi.ac.uk/arrayexpress/experiments/E-GEOD-3307/and for GSE36398 data set, the location is http://0-www.ncbi.nlm.nih.gov.elis.tmu.edu.tw/sites/GDSbrowser?acc=GDS4404. The two datasets differ in the number of columns given that they correspond to different technologies or gene chip versions. Although it is possible to map genes from one technology to another, this process requires a considerable effort that goes beyond the scope of this paper.

The PAMR experiments were conducted through a specific R implementation [39] and run on the same Ubuntu Linux server described above. Specifically, the Nearest Shrunken Centroid classification algorithm works by shrinking each of the class centroids toward the overall centroid by a certain amount called the *threshold*. We used an adaptive computation of this value as provided in the PAMR package.

The SVM-RFE experiments were implemented with the Spider v1.7 software, a MATLAB Machine Learning package popular for feature selection tasks –see http://people.kyb.tuebingen.mpg.de/spider/main.html.
